# Effect of energy restriction and physical exercise intervention on phenotypic flexibility as examined by transcriptomics analyses of mRNA from adipose tissue and whole body magnetic resonance imaging

**DOI:** 10.14814/phy2.13019

**Published:** 2016-11-08

**Authors:** Sindre Lee, Frode Norheim, Torgrim M. Langleite, Hans J. Noreng, Trygve H. Storås, Lydia A. Afman, Gary Frost, Jimmy D. Bell, E. Louise Thomas, Kristoffer J. Kolnes, Daniel S. Tangen, Hans K. Stadheim, Gregor D. Gilfillan, Hanne L. Gulseth, Kåre I. Birkeland, Jørgen Jensen, Christian A. Drevon, Torgeir Holen, the NutriTech Consortium

**Affiliations:** ^1^ Department of NutritionInstitute of Basic Medical SciencesFaculty of MedicineUniversity of OsloOsloNorway; ^2^ Division of CardiologyDepartment of MedicineUniversity of California at Los AngelesLos AngelesCalifornia; ^3^ The Intervention CentreOslo University Hospital OsloOsloNorway; ^4^ Nutrition, Metabolism and Genomics GroupDivision of Human NutritionWageningen UniversityWageningenThe Netherlands; ^5^ Division of Diabetes, Endocrinology and MetabolismDieteticsImperial College Hammersmith CampusLondonUK; ^6^ Research Centre for Optimal HealthDepartment of Life SciencesUniversity of WestminsterLondonUK; ^7^ Department of Physical PerformanceNorwegian School of Sport SciencesOsloNorway; ^8^ Department of Medical GeneticsOslo University HospitalOsloNorway; ^9^ Department of Endocrinology, Morbid Obesity and Preventive MedicineOslo University HospitalOsloNorway; ^10^ Institute of Clinical MedicineFaculty of medicineUniversity of OsloOsloNorway

**Keywords:** Adipose tissue, energy restriction, exercise, immunometabolism, macrophages, obesity

## Abstract

Overweight and obesity lead to changes in adipose tissue such as inflammation and reduced insulin sensitivity. The aim of this study was to assess how altered energy balance by reduced food intake or enhanced physical activity affect these processes. We studied sedentary subjects with overweight/obesity in two intervention studies, each lasting 12 weeks affecting energy balance either by energy restriction (~20% reduced intake of energy from food) in one group, or by enhanced energy expenditure due to physical exercise (combined endurance‐ and strength‐training) in the other group. We monitored mRNA expression by microarray and mRNA sequencing from adipose tissue biopsies. We also measured several plasma parameters as well as fat distribution with magnetic resonance imaging and spectroscopy. Comparison of microarray and mRNA sequencing showed strong correlations, which were also confirmed using RT‐PCR. In the energy restricted subjects (body weight reduced by 5% during a 12 weeks intervention), there were clear signs of enhanced lipolysis as monitored by mRNA in adipose tissue as well as plasma concentration of free‐fatty acids. This increase was strongly related to increased expression of markers for M1‐like macrophages in adipose tissue. In the exercising subjects (glucose infusion rate increased by 29% during a 12‐week intervention), there was a marked reduction in the expression of markers of M2‐like macrophages and T cells, suggesting that physical exercise was especially important for reducing inflammation in adipose tissue with insignificant reduction in total body weight. Our data indicate that energy restriction and physical exercise affect energy‐related pathways as well as inflammatory processes in different ways, probably related to macrophages in adipose tissue.

## Introduction

The increasing incidence of obesity is one of the most important health concerns of the present time. Obesity is closely associated with the development of diabetes, cardiovascular diseases, some forms of cancer and increased mortality (Pi‐Sunyer 1999; Rodriguez et al. 2001; Oliveros and Villamor 2008; Whitlock et al. 2009; Pontiroli and Morabito 2011; Reilly and Kelly 2011; Flegal et al. 2013). A marked increase in the prevalence of obesity has been observed over the past few decades both in industrialized and developing countries (Kelly et al. 2008; Ng et al. 2014).

Obesity is recognized as a chronic low‐grade inflammatory disease, and several studies suggest that inflammation may promote metabolic dysfunction (Schaffler and Scholmerich 2010). Many organs are affected by the low‐grade inflammation such as adipose tissue, pancreas, liver, brain, muscle and intestine. Adipose tissues influence metabolism in distant tissues such as the skeletal muscle, liver, brain and pancreas both by secreting adipokines and by being a major player in lipid metabolism (Stanford et al. 2015).

Adipose tissue includes several cell types such as adipocytes, pre‐adipocytes, fibroblasts, vascular endothelial cells and several different immune cells (Hill et al. 2014). Interestingly, most types of immune cells in adipose tissue changes in number and phenotype in obesity, as shown for T cells linked to obesity‐related metabolic changes (Sell and Eckel 2010; Travers et al. 2015). Most studies the previous decade have, however, been focused on macrophages in adipose tissue (Hill et al. 2014). These adipose tissue macrophages can be classified either as the “M1‐like” or “M2‐like” phenotype, although there is a continuum of different macrophage phenotypes, many of which are not well studied in humans (Hill et al. 2014). M2‐like macrophages contribute to adipose tissue homeostasis by influencing angiogenesis, extracellular matrix remodeling and phagocytosis of adipocytes, whereas M1‐like macrophages promote inflammation in obese adipose tissue, which are related to increased fasting plasma insulin levels, pro‐inflammatory cytokines and insulin resistance (Hill et al. 2014).

Physical activity and energy restriction are major strategies in the prevention and treatment of obesity. Both are known to improve insulin sensitivity (Schenk et al. 2009; Venkatasamy et al. 2013) and reduce adiposity (Swift et al. 2014) and inflammation (Gleeson et al. 2011; Tam and Redman 2013; Lancaster and Febbraio 2014). In addition, these life style changes alter the adipose tissue secretome (Bergmann and Sypniewska 2013) as well as whole body composition. However, the link between fat depots, adipose tissue responses and metabolic flexibility is not fully understood. Thus, for both intervention regimes, energy restriction and enhanced energy expenditure by physical training, we compared the change in individual adipose tissue deposits and ectopic lipid depots in the liver, pancreas and muscle in addition to changes in adipose tissue gene expression.

## Materials and Methods

### Ethical approval

Both studies adhered to the Declaration of Helsinki. The West London Research Ethics committee, London, England, approved the NutriTech study and the National Regional Committee for Medical and Health Research Ethics North, Tromsø, Norway approved the MyoGlu study, which was performed at the Norwegian School of Sport Sciences in Oslo. The two studies were registered with the US National Library of Medicine Clinical Trials registry; NCT01803568 and NCT01684917 for MyoGlu and NutriTech, respectively. Written informed consent was obtained from all participants prior to any study‐related procedure.

### Subjects and exercise intervention

Details and the study protocol regarding the MyoGlu intervention have been published elsewhere (Li et al. 2014; Norheim et al. 2014a,b; Hjorth et al. 2015, 2016; Pourteymour et al. 2015; Langleite et al. 2016). Our current study is focused on the subjects from whom adipose tissue mRNA sequences were available. The subjects were recruited in two groups; an overweight/dysglycemic group (“Exercise”, *n *=* *11, BMI 29 ± 3) and a control group with no dysglycemia (“Exercise, control”, *n *=* *13, BMI 24 ± 2). Dysglycemia was defined as impaired fasting glucose ≥5.6 mmol/L and/or impaired glucose tolerance (2 h serum glucose concentration ≥7.8 mmol/L).

All participants performed 4 h of intense exercise each week, including two whole‐body strength‐training sessions and two spinning bike interval sessions. All exercise were strictly supervised. Strength training sessions started with 10 min aerobic warm‐up followed by three sets of leg press, leg curl, chest press, cable pull‐down, shoulder press, seated rowing, abdominal crunch, and back extension. A linear progression model was used during the intervention. In the three first weeks a load that could be lifted 12 times (12 repetition maximum; RM) was used. In the next 4 weeks 10‐RM was used; and in the remaining weeks 8‐RM were used. For abdominal crunch and back extension 12–20 repetitions were used. Weight loads were increased continuously to obtain the required resistance for the targeted number of repetitions. Endurance exercise included one session of 7 min intervals at 85% of maximum heart rate (HR_max_), and one session of 2 min intervals at 90% of HR_max_ per week. Participants either rested or cycled, with a light load in the periods between the intervals. The period between intervals lasted either 3 min for the 7 min session or 2 min for the 2 min sessions. The number of 7 min intervals were increased from three to four at week 3, and from four to five at week 7. The number of 2 min intervals were increased from 6 to 7 times at week 3, and from 7 to 10 at week 7. The number of attended training sessions and the attendance rate, for both resistance and endurance exercise, for the overweight/dysglycemic and control groups did not differ (*P *>* *0.05).

The diet was monitored by food frequencies questionnaires. All subjects were nonsmoking Caucasian (Langleite et al. 2016).

### Subjects and energy restriction intervention

Details regarding the methodology and the study protocol for the full NutriTech study are currently being prepared for publication. Briefly, the NutriTech included overweight/obese subjects with normal glucose metabolism randomized to either energy restriction (~20% reduced intake of energy from food) (“Diet”, *n = *7, BMI 30 ± 3) or control (“Control”, *n = *5, BMI 30 ± 2) for 12 weeks (Table 1). The diet was monitored by food frequencies questionnaires and 24 h recalls. All subjects were Caucasians.

**Table 1:** Clinical characteristics at baseline and after 12 weeks for the exercise and energy restriction (diet) interventions^a^

	Exercise		Diet		Exercise, control		Control	
	Baseline	12 weeks	Baseline	12 weeks	Baseline	12 weeks	Baseline	12 weeks
Sex (m/f)	11/0		3/4		13/0		3/2	
Age (years)	53 ± 1.7		59 ± 1.3		50 ± 2.1		57 ± 2.7	
GIR (mg/kg/min)^e^	4.2 ± 1.8	5.4 ± 1.8^f^	n.a.	n.a.	7.6 ± 1.6	10.4 ± 2.6^f^	n.a.	n.a.
Weight (kg)	92.9 ± 9.0	91.6 ± 9.1	91.9 ± 17.8	87.3 ± 17.4^f^	78.5 ± 2.3	78.3 ± 2.3	84.7 ± 4.8	84.5 ± 4.9
BMI (kg/m^2^)	28.9 ± 2.5	28.6 ± 2.4	30.8 ± 3.1	29.2 ± 3.0^f^	23.5 ± 2.0	23.5 ± 1.8	29.1 ± 2.0	29.1 ± 2.0
Waist‐hip‐ratio^b^	1.0 ± 0.1	1.0 ± 0.0	0.9 ± 0.1	0.9 ± 0.1	0.9 ± 0.0	0.9 ± 0.0	0.9 ± 0.1	0.9 ± 0.1
Fasting glucose (mmol/L)^d^	5.8 ± 0.4	5.9 ± 0.2	5.0 ± 0.6	4.9 ± 0.6	5.4 ± 0.1	5.5 ± 0.1	4.6 ± 0.3	4.8 ± 0.3
Systolic blood pressure (mmHg)^c^^,^ d	123.0 ± 4.6	129.0 ± 4.2	124.0 ± 9.0	116.0 ± 9.9	117.7 ± 2.9	121.8 ± 2.5	127.6 ± 4.4	122.2 ± 4.7
Diastolic blood pressure (mmHg)^c^^,^ d	75.4 ± 10.2	77.0 ± 6.4	77.6 ± 8.8	64.3 ± 7.7^f^	71.4 ± 1.8	74.0 ± 1.7	78.4 ± 1.9	82.0 ± 5.1
Creatinine (*μ*mol/L)	80.1 ± 7.3	80.5 ± 9.2	74.7 ± 21.3	84.3 ± 13.3	81.6 ± 2.5	80.2 ± 1.8	81.8 ± 5.0	63.8 ± 3.7
ASAT (U/L)	24.6 ± 16.5	18.2 ± 7.2	21.3 ± 3.8	25.1 ± 5.8	18.2 ± 1.8	18.5 ± 1.4	29.2 ± 3.5	25.4 ± 2.4
Total cholesterol (mg/dL)	5.6 ± 0.5	5.8 ± 0.7	5.6 ± 1.2	4.9 ± 1.3^f^	5.1 ± 0.2	4.9 ± 0.1	4.9 ± 0.3	5.1 ± 0.3
HDL‐cholesterol (mg/dL)	1.3 ± 0.2	1.4 ± 0.3	1.5 ± 0.3	1.6 ± 0.2	1.4 ± 0.1	1.4 ± 0.1	1.7 ± 0.1	1.7 ± 0.1
LDL‐cholesterol (mg/dL)	3.6 ± 0.4	3.6 ± 0.6	3.4 ± 1.1	2.6 ± 1.2^f^	3.0 ± 0.2	2.9 ± 0.1	2.7 ± 0.3	2.6 ± 0.4
FFA (10 × mmol/L)	2.2 ± 0.3	2.3 ± 0.2	4.0 ± 0.6	5.4 ± 0.9	2.5 ± 0.4	1.7 ± 0.1^f^	3.2 ± 0.0	2.8 ± 0.0
Leptin (ng/mL)^d^	16.3 ± 6.6	13.5 ± 6.2^f^	16.9 ± 3.2	10.1 ± 3.8^f^	7.9 ± 0.5	7.2 ± 0.5^f^	13.2 ± 3.5	13.2 ± 3.9
IL6 (pg/mL)	2.3 ± 2.9	1.6 ± 1.8	1.8 ± 1.2	1.8 ± 2.3	0.8 ± 0.1	0.8 ± 0.2	0.8 ± 0.8	0.9 ± 0.6
CRP (mg/dL)	3.2 ± 3.5	2.2 ± 2.6	6.6 ± 10.4	3.6 ± .3.0	1.0 ± 0.2	1.3 ± 0.4	1.2 ± 0.8	0.5 ± 0.2

n.a., not available.

a IL, interleukin; CRP, high sensitivity C‐reactive protein; data represent mean ± SD.

b *n* = 9 in the exercise group.

c *n* = 8 in the exercise group.

d *n* = 6 in the diet group.

e GIR; glucose infusion rate measured using the euglycemic hyperinsulinemic clamp.

f *P* < 0.05 versus baseline.

### Blood and tissue sampling

Fasting blood samples and adipose tissue biopsies were taken before as well as after 12 weeks in both interventions. In the exercise intervention, adipose biopsies were taken ~45 min after a standardized acute bicycle challenge (Langleite et al. 2016). The subjects performed 10 min warm‐up and then cycled for 45 min at an individual workload equivalent to 70% of their VO_2max_. VO_2max_ was measured both pre‐ and post intervention to estimate 70% of their VO_2max_ for both the pre‐ and post test (Langleite et al. 2016). Blood sampling in both interventions were performed by standard antecubital venous puncture. Serum‐ and EDTA‐plasma were stored at −80°C until analysis. The subcutaneous adipose tissue biopsies were taken from the periumbilical region and frozen immediately in liquid nitrogen.

### Blood samples analyses

In MyoGlu plasma levels of leptin (Catalog # KAC2281; Invitrogen, Carlsbad, CA) and IL‐6 (Catalog # HS600B; R&D systems, Minneapolis, MN) were measured in duplicates using enzyme‐linked immune‐sorbent assays (ELISA) according to the manufacture's protocols. In NutriTech plasma leptin was measured by ELISA using plates coated with polyclonal anti‐human leptin antibody and a horseradish peroxidase conjugate of the antibody (Catalog # RD191001100; BioVendor, Brno, Czech Republic) and IL‐6 by high sensitivity ELISA (catalog #HEA079Hu; Uscn Life Science Inc., Houston, TX) according to the manufacturer's protocols. Optical density was determined using a micro plate reader (Titertec Multiscan Plus; EFLAB, Helsinki, Finland), set to 450 or 490 nm depending on the actual protocols. Standard curves were generated using best‐fit curves.

### Magnetic resonance imaging and spectrometry

Magnetic resonance (MR) scanning was performed on a 1.5T Philips Achieva MR (Best, The Netherlands) and magnetic resonance spectrometry (MRS) included three Single Voxel Proton Spectroscopy acquisitions (Thomas et al. 1998; Katz et al. 2000; Thomas et al. 2005; Langleite et al. 2016). MR and MRS results were obtained within 3 weeks prior to and 2 weeks after the exercise or diet interventions. Examinations were performed in the evening without any strenuous exercise performed the same day. Total body fat and subcutaneous body fat was measured from ankle‐to‐neck, whereas abdominal body fat was measured in the intra‐ and retroperitoneal spaces. Similar protocols were followed in both interventions.

### Tissue RNA isolation and cDNA synthesis

The protocols used to prepare RNA from adipose tissue biopsies were identical in the MyoGlu and NutriTech studies. Frozen biopsies was transferred into 1 mL QIAzol Lysis Reagent (Qiagen, Hilden, Germany), and homogenized using TissueRuptor (Qiagen) at full speed for 15 sec, twice. Total RNA was isolated from the homogenate using miRNeasy Mini Kit (Qiagen). RNA integrity and concentration were determined using Agilent RNA 6000 Nano Chips on a Bioanalyzer 2100 (Agilent Technologies Inc, Santa Clara, CA). Using High‐Capacity cDNA Reverse Transcription Kit (Applied Biosystems, Foster, CA), 200 ng of total RNA was converted to cDNA for TaqMan real‐time RT‐PCR.

### TaqMan real‐time RT‐PCR

The cDNA reaction mixture was diluted in water and cDNA equivalent of 25 ng RNA used for each sample. Quantitative real‐time PCR was performed with reagents and instruments from Applied Biosystems in the 96‐well format using a 7900HT Fast instrument and the SDS 2.3 software (Applied Biosystems). Predeveloped primers and probe sets (TaqMan assays; Applied Biosystems) were used to analyze mRNA levels of 13 genes for comparison between RT‐PCR, microarray and RNA sequencing. These 13 genes included secreted frizzled‐related protein 4 (*SFRP4*, Hs00180066_m1), leptin (*LEP*, Hs00174877_m1), OPG (*TNFRSF11*, Hs00900358_m1), interleukin‐6 (*IL6*, Hs00985639_m1), adiponectin (*ADIPOQ*, Hs00605917_m1), Apelin (*APLN*, Hs00936329_m1), PR domain containing 16 (*PRDM16*, Hs00922674_m1), T‐box transcription factor (*TBX1*, Hs00271949_m1), trans‐membrane protein 26 (*TMEM26*, Hs00415619_m1), Tumor Necrosis Factor Receptor Superfamily, Member 9 (*TNFRSF9*, Hs00155512_m1), Fibronectin type III domain‐containing protein 5 (*FNDC5*, Hs00401006_m1), Peroxisome Proliferator‐Activated Receptor Gamma, Coactivator 1 Alpha (*PPARGC1A*, Hs01016719_m1), and Uncoupling Protein 1 (Mitochondrial, Proton Carrier) (*UCP1*, Hs00222453_m1). Relative target mRNA expression levels were calculated as 2^−ΔCt^, and normalized to beta‐2 microglobulin (*B2M*, Hs00984230_m1).

### mRNA sequencing

RNA samples were prepared for sequencing using the TrueSeq RNA Sample Prep v2 LS protocol (Illumina, San Diego, CA), employing 1.5 *μ*g input per sample and 4 min fragmentation at 94°C. Samples and their matched controls were ligated to the same indexed adapters and sequenced on separate lanes to minimize any potential adapter ligation or amplification bias. Samples with different indices were blended to equimolar concentrations and sequenced at four samples per lane on a HiSeq 2000 (Illumina). Illumina HiSeq RTA (real‐time analysis) v1.17.21.3 was used for real‐time analysis during the sequencing. Reads passing Illumina's recommended parameters were demultiplexed using CASAVA v1.8.2. For prealignment quality assessments we ran the FastQC v0.10.1 algorithm. The mean library size was ~55 millions unstranded single‐ended reads for adipose tissue with no difference between time points. Base composition in bases 1–12 showed patterns typical for RNA sequencing and bases 13–51 were evenly distributed. All base positions were of high quality (Phred score >30). The biopsies were sequenced in two batches, but no batch effects were detected using clustering analysis. cDNA sequenced reads alignment was performed using Tophat v2.0.8, Samtools v0.1.18, and Bowtie v2.1.0 with default settings against the UCSC hg19 annotated genome dated 14 May 2013 (https://ccb.jhu.edu/software/tophat/igenomes.shtml) and the RefSeqGene transcriptome, which is a project of NCBI's Reference Sequence (RefSeq). Post‐alignment quality checks were done by converting aligned reads to Integrative Genome Viewer v2.3 tracks for visual inspection of normalized signal at any genomic location. BEDtools v2.19.1 was used to calculate coverage. Reads counted by gene feature were performed by the intersection strict mode in HTSeq v0.6.1.

### Microarrays

Purified RNA was labeled with the Affymetrix WT PLUS reagent kit (Affymetrix, Santa Clara, CA) and hybridized to an Affymetrix Human Gene 1.1 ST array plate (Affymetrix). Hybridization, washing, and scanning were carried out on an Affymetrix GeneTitan platform according to manufacturer's instructions. Arrays were analyzed using the R package Oligo (Carvalho and Irizarry 2010) following standard procedures for quality checks and calculation of normalized expression values.

### Pathway analyses

We performed generally applicable gene set enrichment for pathways analysis using GAGE v2.12.3 for global analyses of regulated pathways. The default native workflow was followed as recommended for RNA sequencing and microarray data (Luo et al. 2009).

### Differential gene expression analyses

For differential gene expression analyses of immune cell markers we used the edgeR v3.4.2, DESeq2 v1.4.5 and Cuffdiff v2.1.1 workflows for RNA sequencing data and the LIMMA v3.20.9 workflow for microarray data. Expression levels from RNA sequencing are presented as Reads Per Kilobase of transcript per Million mapped reads and as normalized intensities from microarrays. TaqMan real‐time RT‐PCR was used to evaluate the results from both platforms.

### Markers of adipose tissue macrophages and T cells

The list of macrophage‐specific markers was obtained from studies of human adipose tissue (Capel et al. 2009; Ahlin et al. 2013). Briefly, adipocytes, macrophages, progenitor cells, endothelial cells, and a negative fraction, were separated and analyzed using DNA microarray analyses of the respective cell types. Putative markers showed 1.5–10‐fold higher expression in macrophages compared to the other cell types (Capel et al. 2009). From the putative markers, 18 high confidence markers were defined based on more than twofold higher expression in macrophages as compared to other immune cells in a follow‐up study (Ahlin et al. 2013). The list of M1‐like versus M2‐like macrophage markers in human adipose tissue were chosen based on frequency of use in the literature (Hill et al. 2014). Markers for T cells in human adipose tissue were selected based on the results of Travers et al. (2015). We evaluated the specificity of the markers by comparing the expression in several cells, including immune cells and adipocytes (Lee et al. 2005), in already published data sets obtained from http://www.ncbi.nlm.nih.gov/geo/; GSE3982 and GDS1498. The complete list of markers are available in Table 2. The markers of “metabolically activated” macrophages were taken from the results of Kratz et al. (2014).

**Table 2:** List of markers used in the study

Markers	Symbol	Description	Study
Ma	ACP5	Acid phosphatase 5, tartrate resistant	Capel et al. (2009); Ahlin et al. (2013)
	CCL22	C‐C motif chemokine ligand 22	
	CD68	CD68 molecule	
	CD163	CD163 molecule	
	CHIT1	Chitinase 1	
	CRABP2	Cellular retinoic acid binding protein 2	
	CSF1R	Colony stimulating factor 1 receptor	
	GLA	Galactosidase alpha	
	GM2A	GM2 ganglioside activator	
	IL1RN	Interleukin 1 receptor antagonist	
	LILRB4	Leukocyte immunoglobulin like receptor B4	
	LIPA	Lipase A, lysosomal acid type	
	MRC1	Mannose receptor, C type 1	
	MSR1	Macrophage scavenger receptor 1	
	PLA2G7	Phospholipase A2 group VII	
	PLA2G15	Phospholipase A2 group XV	
	SIGLEC1	Sialic acid binding Ig like lectin 1	
	SLC38A6	Solute carrier family 38 member 6	
M1‐like	CCL2	C‐C motif chemokine ligand 2	Hill et al. (2014)
	TNF	Tumor necrosis factor	
	IL8	Interleukin 8	
	COX20	COX20 cytochrome c oxidase assembly factor	
	IL6	Interleukin 6	
	IL1B	Interleukin 1 beta	
	ITGAX	Integrin subunit alpha X	
	TLR4	Toll like receptor 4	
	CCR2	C‐C motif chemokine receptor 2	
	IL1RN	Interleukin 1 receptor antagonist	
M2‐like	IL10	Interleukin 10	
	MRC1	Mannose receptor, C type 1	
	TGFB1	Transforming growth factor beta 1	
	CCL18	C‐C motif chemokine ligand 18	
	CD163	CD163 molecule	
	ITGB5	Integrin subunit beta 5	
T cells	CD3E	CD3e molecule	Travers et al. (2015)
	CD4	CD4 molecule	
	CD8A	CD8a molecule	
	TBX21	T‐box 21	
	GATA3	GATA binding protein 3	
	FOXP3	Forkhead box P3	
MAM	ABCA1	ATP‐binding cassette transporter 1	Kratz et al. (2014)
	PLIN2	Perilipin 2	

Ma, macrophage markers; MAM, Metabolically activated macrophages.

### Statistical analyses

Parametric Welch *t*‐tests were performed and the results were comparable to non‐parametric Wilcoxon signed‐rank tests, ensuring no discrepancies in conclusions depending on the statistical test (not included). Correlations were analyzed between the mean expression of macrophage markers and the variables presented in Table 1 at baseline and between changes. Pearson's or Spearman's correlations existing in both study groups are presented. Fold‐changes are presented in log_2_, which are symmetrical around 0, that is, 2.0‐fold (1.0 in log_2_) and 0.5‐fold (−1.0 in log_2_) is presented equal in magnitude, and <0.0 represents down‐regulation whereas >0.0 represents up‐regulation. Two‐way tests were performed and statistical significance was defined as *P < *0.05. Statistical evaluations were performed in R v3.0.3.

## Results

### Group comparability

The exercise intervention increased energy expenditure by ~17% and the diet intervention reduced energy intake by ~18% as estimated based on data from Mifflin et al. (1990) in lack of more direct methods. At baseline, all subjects had a physical activity level of 1.2 (defined as little or no exercise) and the exercise intervention increased this to 1.55 (defined as moderate exercise 3–5 days a week). Total energy expenditure in the exercise group was ~15,800 kcal/week, which increased to ~18,400 kcal during the intervention corresponding to ~17% increase. Total energy expenditure in the diet group was ~14,500 kcal/week and the loss in body weight (three forth being adipose tissue and one forth being from fat free mass based on data from Heymsfield et al. (2014)) corresponding to ~2600 kcal/week, which equals to ~18% decrease.

The two groups were also comparable in regards to age (~50–60 years), BMI, included only Caucasians, were of equal duration and used similar protocols. However, the exercise group included only dysglycemic men, whereas the energy restriction group were mixed‐sex with no dysglycemia.

### Metabolic markers and adipose tissue depots

Both interventions were successful as reflected in increased insulin sensitivity (glucose infusion rate measured with the hyperinsulinemic‐euglycemic clamp) after exercise of 36% and 29% in the overweight/obese and control subjects respectively, and 5% weight loss in the energy restriction group (Table 1). The exercise groups had insignificant reductions in body weight (1.4%, *P *>* *0.05 and 0.2%, *P *>* *0.05 for the overweight/obese and control subjects, respectively) although body composition changed markedly (Langleite et al. 2016). Blood plasma cholesterol levels were improved in both interventions, reflected in increased high‐density lipoprotein (HDL) cholesterol after exercise and reduced HDL cholesterol after energy restriction (Table 1). Moreover, circulating leptin concentration was reduced in both interventions (Table 1).

Total amount of adipose tissue, subcutaneous adipose tissue and intra‐abdominal adipose tissue were reduced in both cohorts (Table 3). Furthermore, reduced hepatic fat content was observed in both cohorts, seemingly to a larger extent after exercise (Table 3).

**Table 3:** Changes in fat depots after 12 weeks exercise and energy restriction (diet)^a^

	Exercise	Diet^b^	Exercise, control	Control^b^
	Change (%)	Change (%)	Change (%)	Change (%)
MRI				
Total AT	−10.9 ± 5.1^f^	−8.9 ± 3.3^f^	−8.5 ± 2.7^f^	3.7 ± 1.4
Subcutaneous AT	−7.3 ± 6.0^f^	−8.6 ± 3.7^f^	−6.6 ± 2.6^f^	2.0 ± 1.0
Intra‐abdominal AT	−19.4 ± 10.8^f^	−11.4 ± 6.2^f^	−16.9 ± 4.2^f^	7.7 ± 3.3
MRS fat				
Pancreas^c^	−28.5 ± 62.9	−20.8 ± 49.7	−30.3 ± 21.7	21.3 ± 20.6
Liver^d^	−27.4 ± 15.7^f^	−7.4 ± 2.4^f^	−23.3 ± 14.1^e^	−6.8 ± 1.9^f^

a Data represent mean ± SEM. Only relative values are presented due to slight differences in protocols and units calculated in the two cohorts.

b Only data from six subjects in the diet group were available.

c *n* = 7 in the exercise group.

d *n* = 9 in the exercise group.

e The reduction in the control group is significant using the Wilcoxon test (Langleite et al. 2016).

f *P* < 0.05 (baseline vs. 12 weeks).

### mRNA platform evaluation

**Figure 1:**
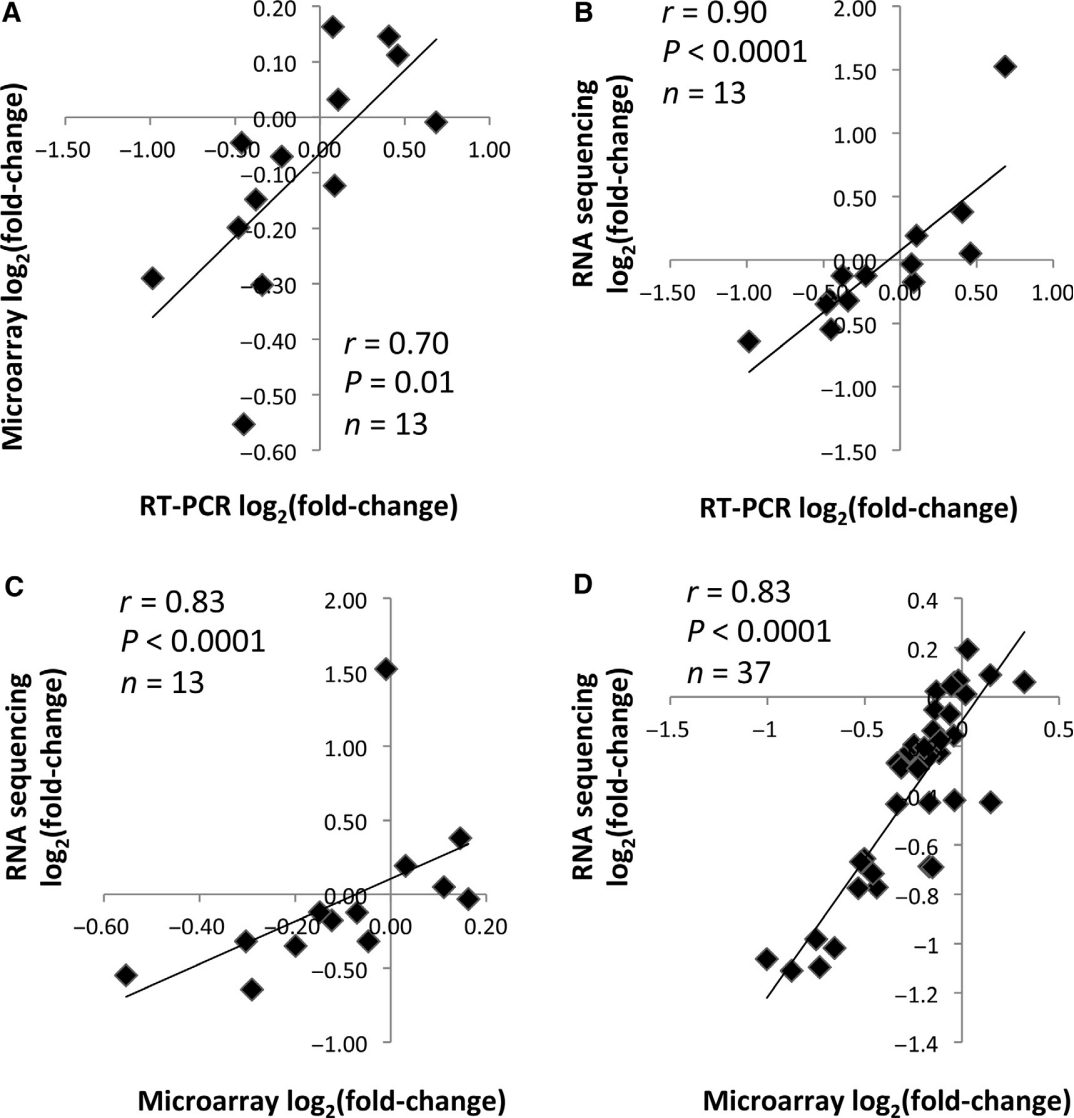
Correlations between mRNA sequencing, microarray and RT‐PCR. (A) Correlation between the change in expression of 13 genes (see “Methods”) analyzed by RT‐PCR and microarray. (B) Correlation between the change in expression of 13 genes analyzed by RT‐PCR and mRNA sequencing. (C) Correlation between the change in expression of 13 genes analyzed by microarray and mRNA sequencing. (D) Correlation between the change in expression of 37 immune cell markers (see Table 2) analyzed by microarray and mRNA sequencing. The change (post baseline) was calculated from the average expression from five subjects from the exercise intervention. Spearman's rho correlations were performed.

We evaluated how RNA sequencing, microarray and RT‐PCR compared in regards to change in gene expression. Thirteen genes were analyzed by all three platforms from a subset of the subjects from the exercise intervention (*n* = 5) (Fig. 1A–C). In addition, the change in immune cell markers were compared between RNA sequencing and microarray (Fig. 1D).

### Altered pathways after exercise and energy restriction

We limited our analysis to pathways altered after both interventions. These included pathways related to immunity and energy metabolism (Table 4). Interestingly, immune‐related and energy‐related pathways were regulated in opposite directions after exercise and energy restriction. Immune‐related pathways were reduced and energy‐related pathways were increased after exercise, whereas the opposite was observed after energy restriction with increased immune‐related pathways and reduced energy‐related pathways (Table 4). No alterations in these pathways were observed in control subjects from the exercise or diet interventions (not included).

**Table 4:** Enrichment analysis of pathways altered in adipose tissue after exercise and energy restriction (diet)^a^

	Exercise		Diet	
	Up/down	*P‐*value	Up/down	*P‐*value
Immune‐related pathways				
Chemokine signaling pathway	−5.1	2.4E‐07	1.7	6.0E‐06
Osteoclast differentiation	−5.4	7.5E‐08	2.1	2.7E‐08
Complement and coagulation cascades	−3.3	6.3E‐04	1.3	4.5E‐03
Toll‐like receptor signaling pathway	−3.8	1.0E‐04	1.9	3.0E‐03
NOD‐like receptor signaling pathway	−2.5	7.4E‐03	1.0	6.4E‐03
Jak‐STAT signaling pathway	−2.3	1.1E‐02	1.2	9.2E‐03
Hematopoietic cell lineage	−4.1	2.7E‐05	2.7	2.1E‐12
Natural killer cell mediated cytotoxicity	−5.4	8.3E‐08	2.6	6.5E‐12
T‐cell receptor signaling pathway	−3.4	3.8E‐04	1.9	3.1E‐07
B‐cell receptor signaling pathway	−4.1	3.2E‐05	1.3	2.7E‐04
Leukocyte transendothelial migration	−4.7	1.8E‐06	1.3	2.1E‐04
Energy‐related pathways				
Glycolysis/gluconeogenesis	0.5	0.040	−8.4	0.014
Citrate cycle (TCA cycle)	0.9	0.002	−1.6	3.7E‐05
Fatty acid metabolism	0.9	0.002	−0.9	7.0E‐03
Alanine, aspartate and glutamate metabolism	0.5	0.047	−0.8	0.019
Pyruvate metabolism	0.7	0.013	−1.1	0.004
Peroxisome	0.6	0.031	−1.2	9.2E‐04
Insulin signaling pathway	0.7	0.011	−0.8	0.014

a Pathways are listed by Kyoto Encyclopedia of Genes and Genomes database (KEGG, R database v.2.14.0) nomenclature for homo sapiens. Enrichment analyses were performed using the R package GAGE v.2.14.4, which is applicable independent of microarray or RNA sequencing data attributes including sample sizes, experimental designs, assay platforms, and other types of heterogeneity. The table depicts pathways enriched with a significance value of *P* < 0.05 after exercise or energy restriction. “Up/down” represents the GAGE estimate of a pathway being up (positive) or down (negative) regulated.

### Adipose tissue macrophages

Adipose tissue macrophages play important roles in regulation of inflammation and metabolism in adipose tissue (Hill et al. 2014). We expected effects on adipose tissue macrophages based on the observed changes in immunity‐ and energy‐related pathways in both interventions. Thus, we monitored 18 markers of human adipose tissue macrophage.

**Figure 2:**
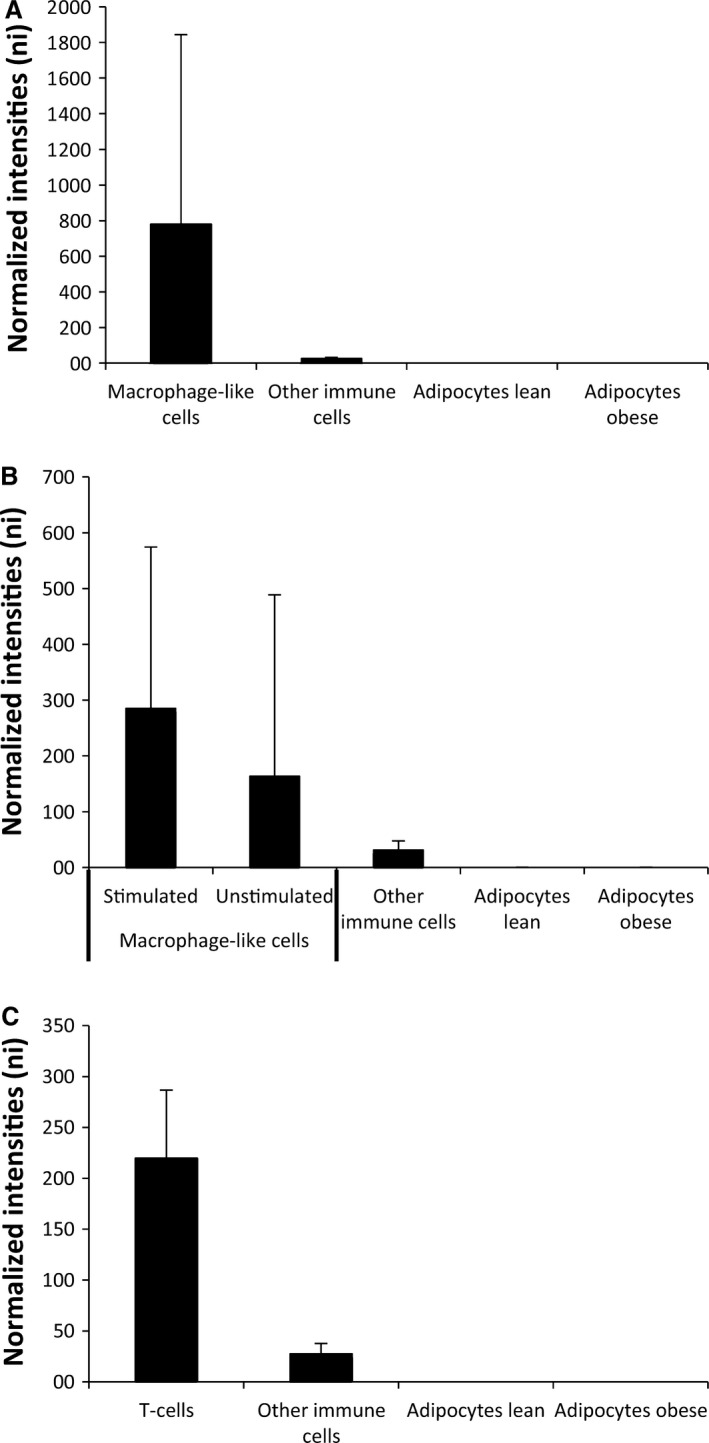
Markers of immune cell subtypes. Expression of the 37 markers of cell subtypes (see “Table 2”) in human adipose tissue. (A) The median expression of 18 markers of adipose tissue macrophages compared across the median of macrophage‐like cells (*n* = 12), other immune cells (*n* = 22), lean adipocytes (*n* = 20) and obese adipocytes (*n* = 19). (B) The median expression of 16 markers of M1‐like and M2‐like macrophages compared across the median of activated macrophage‐like cell (*n* = 6), inactivated macrophage‐like cells (*n* = 6), other immune cells (*n* = 22), lean adipocytes (*n* = 20), and obese adipocytes (*n* = 19). (C) The median expression of six markers of T cells compared across the median of T cells (*n* = 10), other immune cells (*n* = 26), lean adipocytes (*n* = 20), and obese adipocytes (*n* = 19). The full expression panel for every marker in every cell type is available in Figure 3. Data are medians + interquartile range.

**Figure 3:**
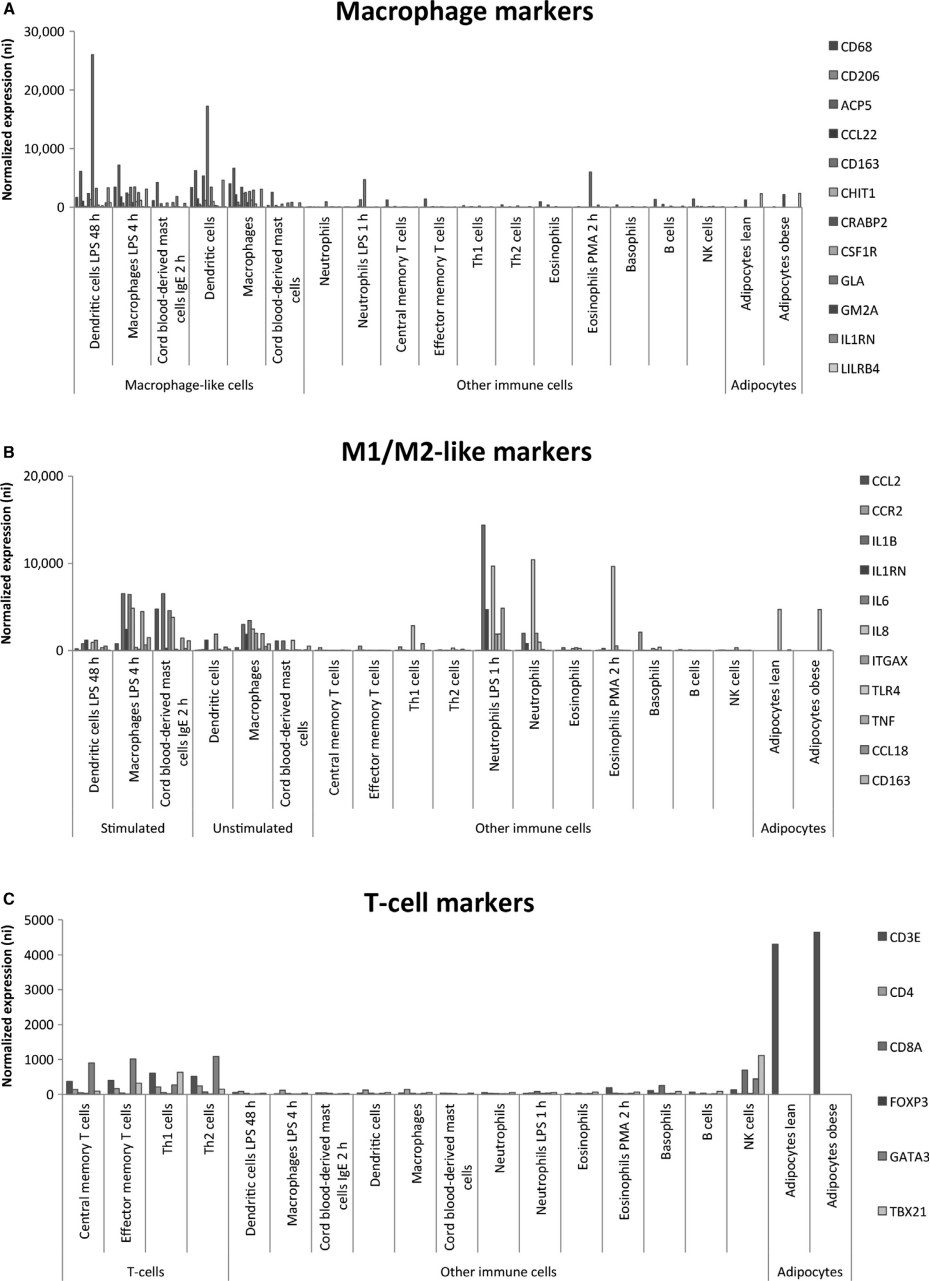
Expression of mRNA markers of macrophages, M1‐ and M2‐like macrophages and T cells in human adipose tissue. (A) Expression of the 18 markers of macrophages in macrophage‐like cells compared to the expression in other immune cells and adipocytes. (B) Expression of the 11 markers of M1/M2‐like macrophages in stimulated macrophage‐like cells compared to the expression in unstimulated macrophage‐like cells, other immune cells and adipocytes. (C) Expression of six markers of T cells in T cells compared to expression in other immune cells and adipocytes.

First, we detected higher expression of the markers in macrophage‐like cells (Lipopolysaccharide [LPS]‐stimulated macrophages, LPS‐stimulated dendritic cells and IgE‐stimulated cord blood‐derived mast cells) compared to other immune cells, and adipocytes from both lean and obese subjects (Fig. 2A) as published elsewhere (Lee et al. 2005; Ahlin et al. 2013; Hill et al. 2014; Travers et al. 2015). For more details see Figure 3. Furthermore, at least two proteins encoded by the markers (CD208 and CD68) colocalize to human adipose tissue macrophages in “crown‐like structures” (Moreno‐Navarrete et al. 2013).

**Figure 4:**
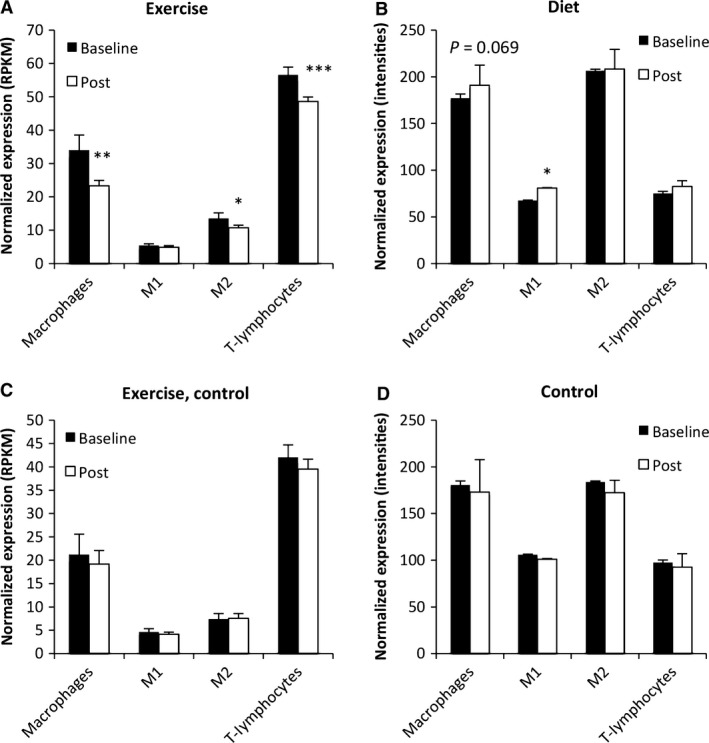
The change in expression of immune cell markers in response to exercise and diet. (A) The average expression of markers for adipose tissue macrophage, M2‐like macrophages and T cells were reduced after exercise. (B) The average expression of markers for adipose tissue M1‐like macrophages was increased after energy restriction. (C) No alteration in expression of immune cell markers was observed in lean control subjects after exercise. (D) No alteration in expression of immune cell markers was observed in control subjects. Data represent means ± SEM. *P < 0.05, **P < 0.01, and ***P < 0.001 baseline versus 12 weeks. RPKM = Reads Per Kilobase of transcript per Million mapped reads; ni = normalized intensities.

**Figure 5:**
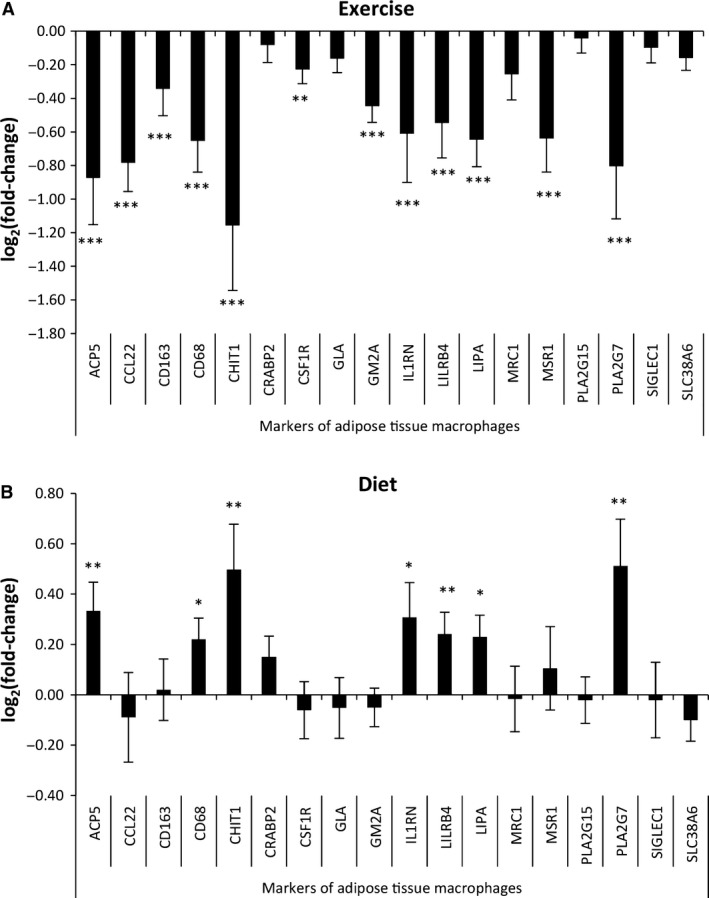
Opposite regulation of adipose tissue macrophages after exercise versus energy restriction. (A) Reduced expression (log_2_[fold‐change] < 0) of 12 of 18 markers of adipose tissue macrophages was observed after exercise. (B) Increased expression (log_2_[fold‐change] > 0) of seven of 18 markers of adipose tissue macrophages was observed after energy restriction. Data represent means ± SEM. *P < 0.05, **P < 0.01, and ***P < 0.001.

**Figure 6:**
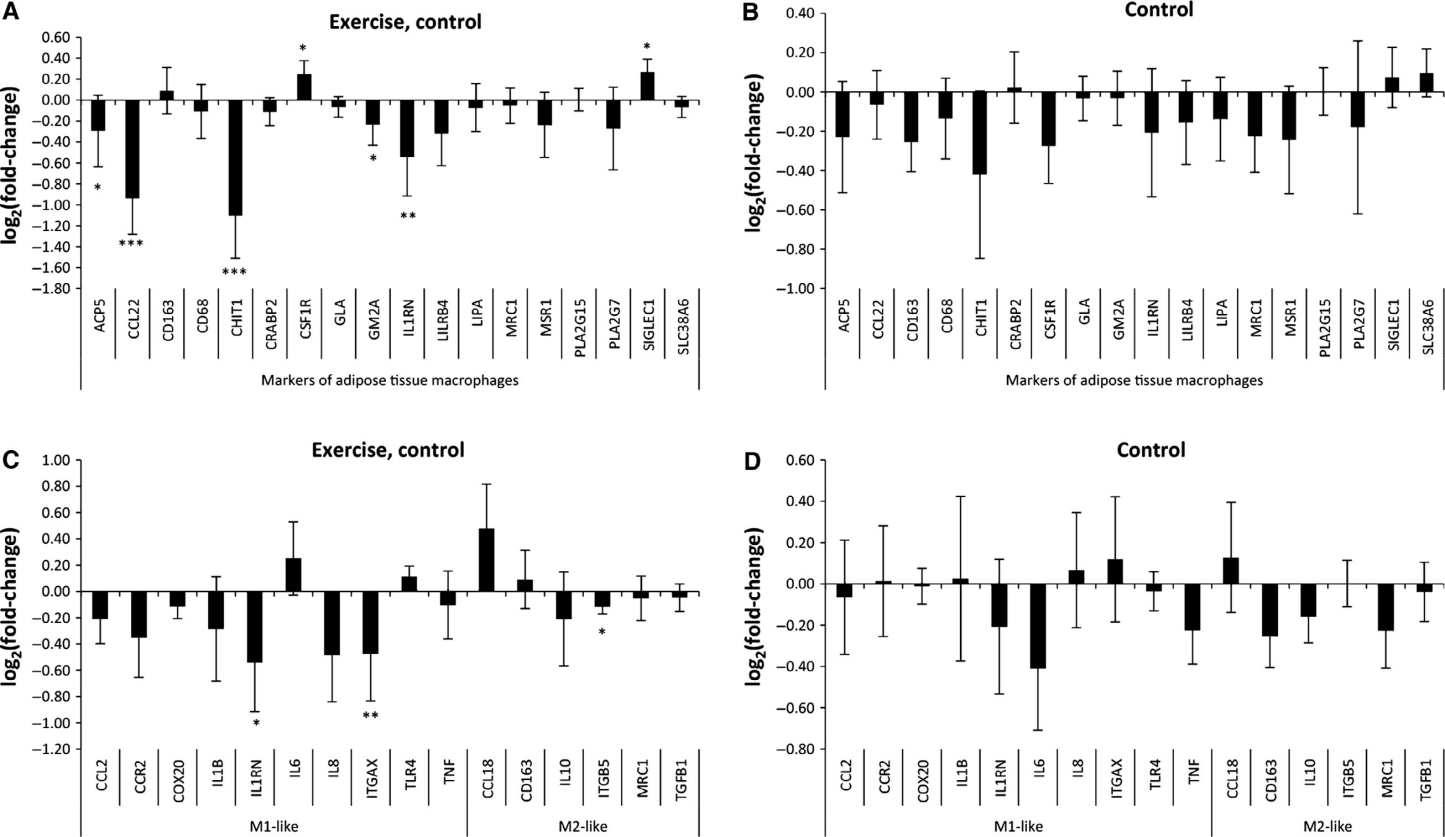
Expression of macrophage markers in control subjects. (A) No consistent change in expression of 18 markers of macrophages was observed after exercise in lean control subjects. (B) No change in expression of 18 markers of macrophages was observed in control subjects. (C) No consistent change in expression of 11 markers of M1‐ and M2‐like macrophages was observed after exercise in lean control subjects. (B) No change in expression of 11 markers of M1‐ and M2‐like macrophages was observed in control subjects. Data represent means ± SEM. *P < 0.05, **P < 0.01 and ***P < 0.001.

Second, a marked down‐regulation of macrophage markers was observed after exercise (Fig. 4A). The expression of all 18 markers was reduced after exercise, of which 12 reached statistical significance (Fig. 5A). In contrast, a trend toward down‐regulation of macrophage markers was observed after energy restriction (Fig. 4B). The expression of seven of 12 markers had enhanced expression and reached statistical significance (Fig. 5B). No clear alteration in expression of macrophage markers were observed for control subjects in the exercise intervention (Figs. 4C and 6A) and in the diet intervention (Figs. 4D and 6B).

### Adipose tissue macrophage phenotypes

Macrophages include several cell types that for the sake of simplicity can be classified into M1‐like “classically” activated macrophages and M2‐like “alternatively” activated macrophages (Hill et al. 2014).

To address if any changes with regard to macrophage phenotypes had occurred due to the interventions, we evaluated markers of the M1‐ and M2‐like phenotypes. First, we observed higher expression of the markers in activated macrophage‐like cells compared to other cells types from the innate and adaptive immune system and adipocytes from lean and obese subjects *in vivo* (Fig. 2B). LPS‐stimulated neutrophils also expressed some of these markers (Fig. 3).

**Figure 7:**
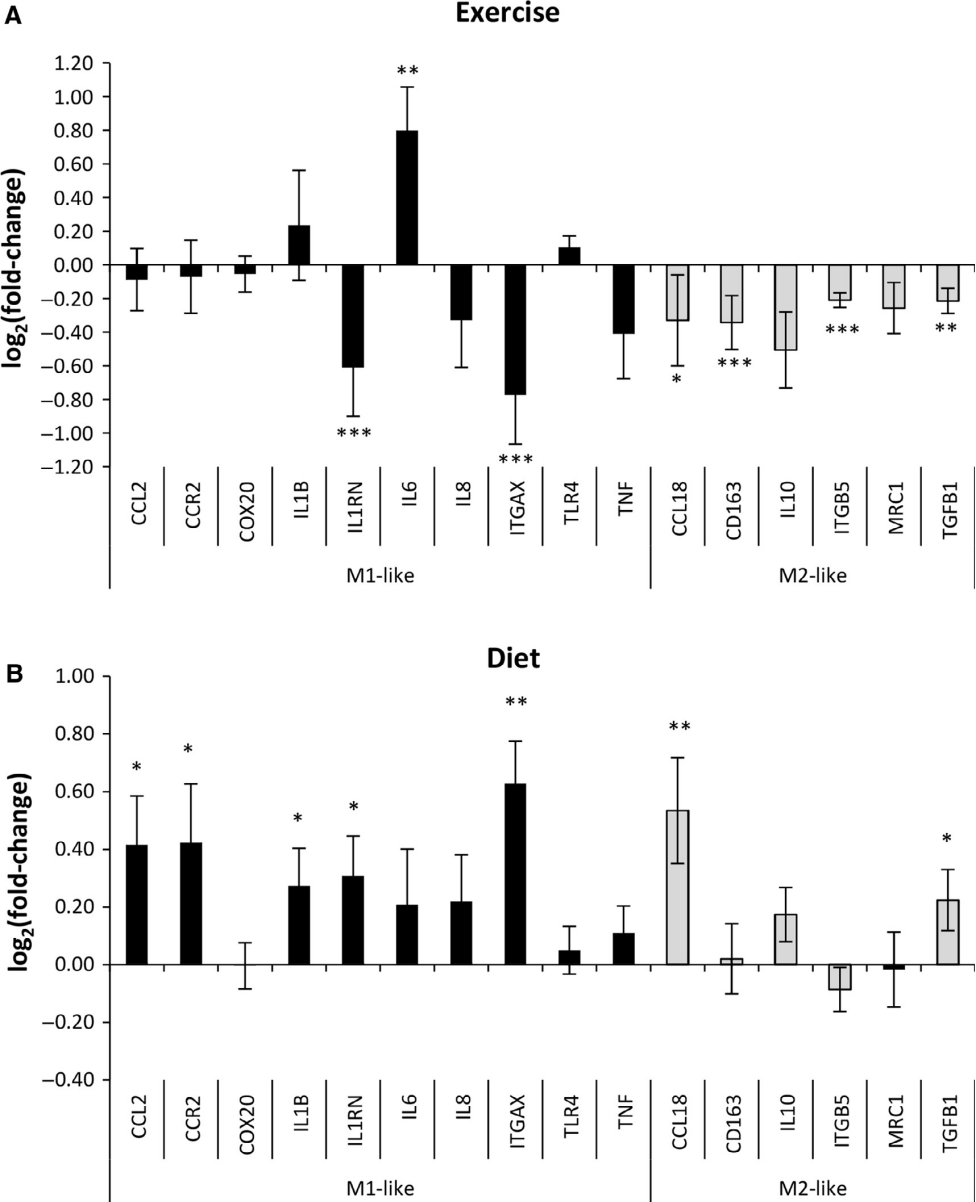
Exercise and diet influence adipose tissue macrophage subtypes differently. (A) Reduced expression (log_2_[fold‐change] < 0) of 4 out of 6 markers of adipose tissue M2‐like macrophages was observed after exercise. (B) Increased expression (log_2_[fold‐change] > 0) of five of 10 markers of adipose tissue M1‐like macrophages was observed after energy restriction. Data represent means ± SEM. *P < 0.05, **P < 0.01 and ***P < 0.001.

The expression of M2‐like markers was reduced after exercise in overweight/obese subjects (Figs. 4A and 7A). The expression of M1‐like markers was increased after energy restriction in overweight/obese subjects (Figs. 4B and 7B). No marked alteration in expression of macrophage M1/M2‐like markers were observed for control subjects in the exercise intervention (Figs. 4C and 6A) and in the diet intervention (Figs 4D and 6B).

**Figure 8:**
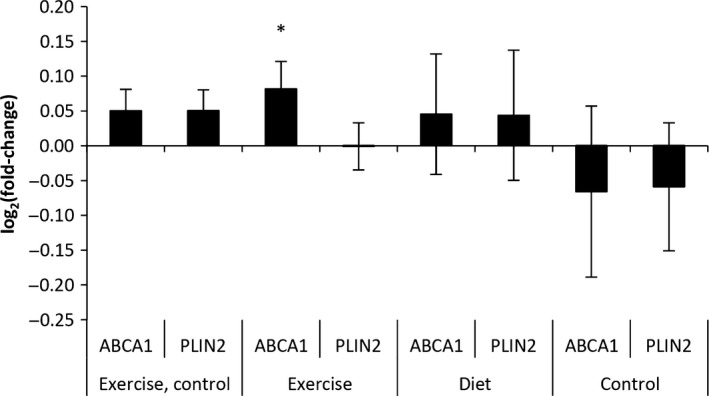
mRNA expression of markers of “metabolically activated” macrophages. No consistent change in two markers of “metabolically activated” macrophages were observed in neither the exercise nor in the diet intervention. Data represent means ± SEM. *P < 0.05.

We did not observe any marked alterations in expression of the two markers of “metabolically activated” macrophages (Fig. 8).

### Adipose tissue T cells

Adipose tissue contains various types of immune cells in addition to macrophages (Sell and Eckel 2010). T cells are known to have several roles in adipose tissue including interactions with adipose tissue macrophages (Hill et al. 2014), regulation of macrophage phenotypes and adipocyte function (Sell and Eckel 2010; Travers et al. 2015). Thus, we analyzed markers of adipose tissue T cells.

First, we observed higher expression of the markers in T cells compared to other immune cells and adipocytes from lean and obese subjects (Fig. 2C). For more details see Figure 3. Second, the expression of T‐cell markers was reduced after exercise in overweight/obese subjects (Fig. 2A). No alteration in the expression of T‐cell markers was observed in the diet intervention (Fig. 4B and D) and in control subjects in the exercise intervention (Fig. 4C).

### Macrophages, adiposity and metabolic markers

**Figure 9:**
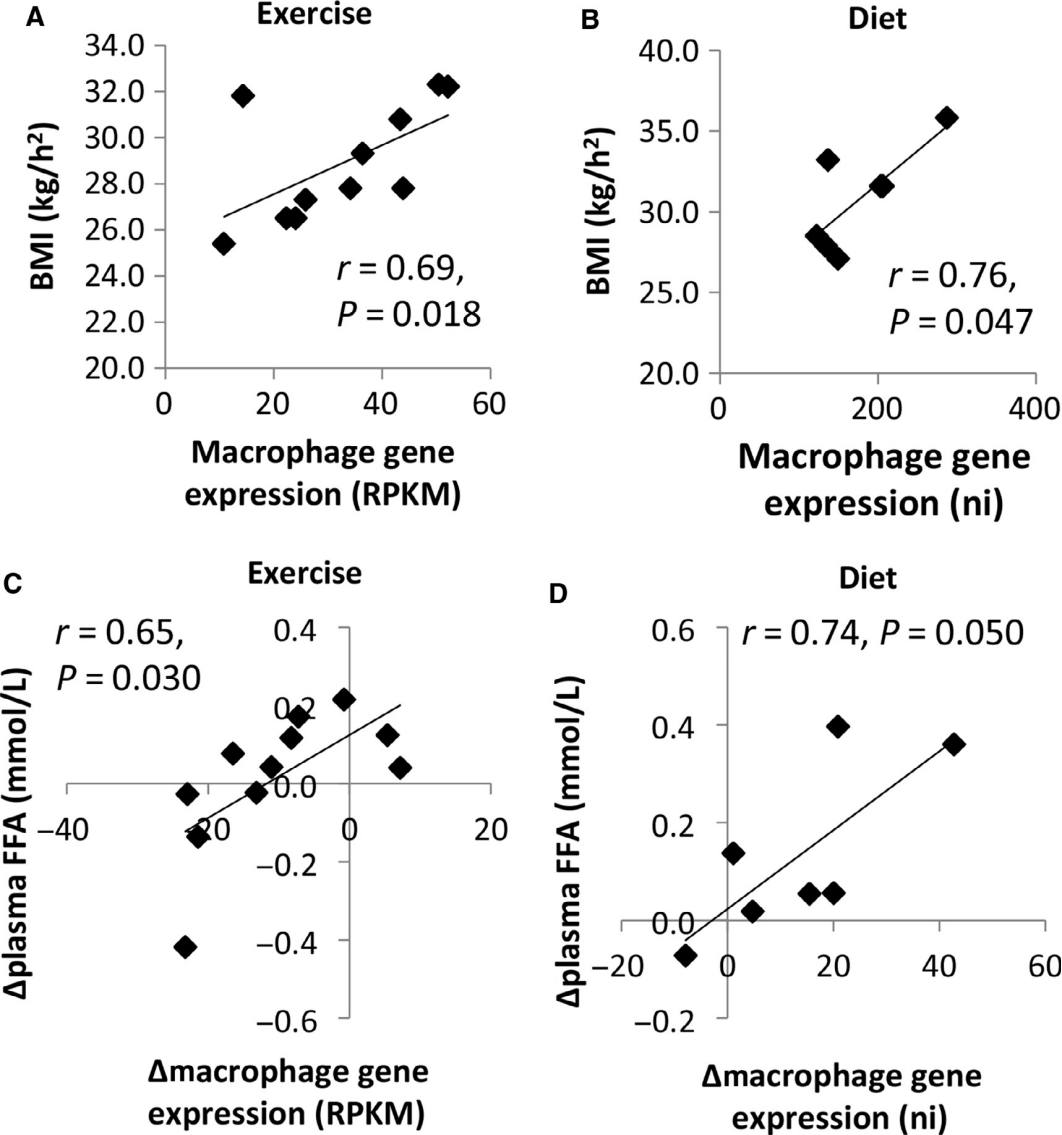
Macrophage markers correlate with BMI and FFA in both the exercise and diet groups. (A) BMI correlated with adipose tissue macrophage expression at baseline in the exercise group. (B) BMI correlated with adipose tissue macrophage expression at baseline in the diet group. (C) The change in gene expression (12 weeks minus baseline) of adipose tissue macrophages correlated with the change in plasma FFA concentrations in the exercise group. (D) The change in gene expression of adipose tissue macrophages correlated with the change in plasma FFA concentrations in the diet group. RPKM = Reads Per Kilobase of transcript per Million mapped reads, ni = normalized intensities.

We performed correlation analyses on baseline values and longitudinal changes (data after 12 weeks intervention minus baseline) in regard to adipose tissue macrophage expression against T‐cell expression and the variables presented in Table 1. Only statistically significant correlations observed in both study populations were emphasized; correlation between BMI and adipose tissue macrophages at baseline (Fig. 9A–B) and correlation between changes in plasma concentration of free fatty acids (FFA) and changes in adipose tissue macrophages (Fig. 9C–D). The most marked correlation was between T‐cell expression and adipose tissue macrophage expression. These correlations were observed both at baseline (*r* = 0.89, *P *<* *0.001 and *r* = 0.89, *P *<* *0.01) and longitudinally (*r* = 0.78, *P *<* *0.01 and *r* = 0.71, *P *=* *0.08) in both types of interventions.

## Discussion

Our main finding was that interventions with exercise and energy restriction influenced immune‐ and energy‐related mRNA expression in adipose tissue in opposite ways in overweight/obese subjects, despite having similar beneficial effects on metabolic markers and adipose tissue depots. We did not observe these alterations in control subjects in the exercise or the diet interventions.

The reduction in abdominal obesity, visceral fat and liver fat depots in subjects with overweight/obesity suggests important improvement in health risk factors. These fat depots are associated with increased insulin resistance, glucose intolerance, high blood lipids, cardiovascular disease and mortality (Frayn et al. 1992). These co‐morbidities also contribute to development of atherosclerotic disease through effects on plaque formation, platelet aggregation, and smooth muscle proliferation (Yki‐Jarvinen and Westerbacka 2000). The reduction in hepatic fat deposition in both interventions are particularly interesting because it may be an independent predictor of insulin resistance, type II diabetes and mortality (Kuk et al. 2006; Sattar and Gill 2014). The accumulation of triglycerides in hepatocytes, in the absence of alcohol abuse, can lead to nonalcoholic fatty liver disease. This disease is a common condition that can develop into steatohepatitis, cirrhosis, and liver‐related death (Paschos and Paletas 2009). On average liver fat is fourfold higher in subjects with the metabolic syndrome and correlates significantly with all components of the syndrome, including visceral fat and serum triglycerides and inversely with HDL (Chai et al. 2016). Furthermore, our data suggest a reduction in pancreatic fat content in the intervention groups, which is interesting because ectopic pancreatic lipid in the pancreas may cause *β*‐cell failure (Shimabukuro et al. 1998). Pancreatic fat content has been found to be significantly higher in diabetic patients compared with controls (Chai et al. 2016). In addition, the degree of steatosis in the nondiabetic group negatively correlated with *β*‐cell function parameters (Kantartzis et al. 2006). However, the variations in effects we observed in this study were extensive, probably due to interference with surrounding omental fat.

Adipose tissue inflammation and metabolism are closely related and have been referred to as “immunometabolism” (Hill et al. 2014). Key players in the regulation of both inflammation and metabolism include adipose tissue macrophages as well as various types of immune cells from the innate as well as the adaptive immune system (Sell and Eckel 2010). In the case of obesity, enhanced amounts of body fat increases the number of adipose tissue macrophages (Bouloumie et al. 2008) and induces chronic low‐grade inflammation (Wysocki et al. 2005). T cells precede macrophages in adipose tissue inflammation and may influence macrophage phenotypes and adipocyte function (Sell and Eckel 2010; Travers et al. 2015). T cells may alter immune function in adipose tissue already at slight adiposity (Travers et al. 2015).

### Energy restriction

Increased immune‐related gene expression after 5% weight loss has been observed in obese women and men (Capel et al. 2009; Magkos et al. 2016). Our new observations demonstrate increased M1‐like adipose tissue macrophages. Although increased adipose tissue macrophages might seem unwanted, our results suggest that plasma FFA levels may be important contributors to this increase. Serum FFA concentrations correlate with total rates of lipolysis and fatty acid fluxes in adipose tissue (Duncan et al. 2007). Increased lipolysis and high levels of local lipid fluxes are associated with increased numbers of adipose tissue macrophages after energy restriction. Local lipid flux may influence adipose tissue macrophages recruitment and initiate formation of lipid‐laden macrophages capable of buffering local increase in lipid concentration (Kosteli et al. 2010). In mice, increased numbers of adipose tissue macrophages were observed acutely after energy restriction and correlated with serum FFA levels (Kosteli et al. 2010). Six of seven of our participants increased plasma FFAs after energy restriction (Fig. 9D). Furthermore, pro‐inflammatory (M1‐like) macrophages have a greater lipid content compared to anti‐inflammatory (M2‐like) macrophages in diet‐induced obesity (Prieur et al. 2011). This suggests that mostly M1‐like adipose tissue macrophages accumulate excess lipids in adipose tissue. However, it might also imply that lipid accumulation promotes accumulation of M1‐like adipose tissue macrophages.

After prolonged energy restriction reduced adipose tissue mass and adipocyte size are observed. This leads to reduced basal lipolysis and reduced numbers and/or activity of adipose tissue macrophages both in humans (Magkos et al. 2016) and rodents (Vieira et al. 2009a). Inflammation‐related gene expression increased at 5% weight loss, but was reduced after 16% weight loss compared to baseline in humans (Magkos et al. 2016). Coherent with this observation, 17% weight loss in patients 12 weeks after bariatric surgery reduced adipose tissue inflammation (Clement et al. 2004; Cancello et al. 2005). We observed increased activity of M1‐like adipose tissue macrophages after 5% weight loss. This might reflect the short period of energy restriction and/or the modest weight loss. A longer intervention, and/or greater weight loss, would probably result in a reduction.

### Exercise

Most studies on exercise and adipose tissue inflammation are performed on mice fed a high fat diet (HFD). In male, C57BL/6 mice after consuming a HFD for 4, 12 weeks exercise reduced adipose tissue inflammation and adiposity measured by gene expression of circulating amyloid A, adipose F4/80, MCP‐1, and TNF‐*α* (Samaan et al. 2014). Similar results were observed in Balb/cByJ mice fed a HFD. Reduced adipose tissue mRNA expression of MCP‐1 was observed after exercise (Vieira et al. 2009b). These results are in agreement with another HFD study on C57BL/6J male mice, where expression of MCP‐1, F4/80, and neutrophil elastase were reduced in adipose tissue after exercise (Kawanishi et al. 2015). In addition to reduced adipose tissue macrophages, Kawanishi et al. suggested that exercise also reduced neutrophil infiltration, which precedes macrophage infiltration and adipose tissue inflammation. The mRNA markers of macrophages are also highly expressed in activated neutrophils (Fig. 3). Thus, these markers should not be interpreted as specific adipose tissue macrophage markers. Furthermore, in male C57BL/6 mice consuming a HFD, exercise inhibited mRNA expression of TNF‐alpha, ICAM‐1, and F4/80 in adipose tissue without reduction in adipose tissue mass (Kawanishi et al. 2010).

Although M1‐like adipose tissue macrophages tend to organize in crown like structures (Eguchi and Feldstein 2014), M2‐like adipose tissue macrophages are associated with fibrosis, correlating negatively to insulin sensitivity, and are found in high numbers in insulin‐resistant humans (Spencer et al. 2010). The exact roles of M2‐like adipose tissue macrophages are unknown, but at least in mice they produce anti‐inflammatory mediators such as IL‐10, and may play a critical role in the maintenance of adipose tissue insulin sensitivity (Lumeng et al. 2007). Thus, M2‐like adipose tissue macrophages might be increased in insulin resistant humans as a compensatory mechanism. We observed reduced expression levels of markers for adipose tissue macrophages and the M2‐like phenotype after exercise. This indicates reduced amounts of adipose tissue macrophages with the M2‐like phenotype perhaps related to increased insulin sensitivity after exercise and reduced need of the compensatory mechanism.

We also observed a reduction in adipose tissue mRNA markers of T cells after exercise. T cells orchestrate inflammatory processes in adipose tissue and liver. Costimulatory molecules are known to mediate cross‐talk between the adaptive and innate immune system and to direct T‐cell responses in inflammation (Seijkens et al. 2014). Our data suggest that adipose tissue T cells mediate some of the beneficial effects of exercise.

### Strengths and limitations

Limitations in this study include difference in sex composition between the two interventions (all male vs. mixed‐sex), a limited sample size, dysglycemic metabolism in overweigh/obese subjects from the exercise intervention and normal glucose metabolism in overweigh/obese subjects from the diet intervention, differences in the biopsy time points in the two interventions and a lack of more direct measures of immune cells in adipose tissue and energy expenditure.

We have not distinguished sex‐specific responses to energy restriction due to the limited sample size. Moreover, dysglycemia might induce changes in adipose tissue, such as increased fibrosis, which is associated with increased amounts of M2‐like macrophages (Spencer et al. 2010). The different alterations in markers of macrophages in the exercise and diet interventions might be cofounded by differing baseline states concerning glucose metabolism. Furthermore, the biopsies were taken 45 min after a standardized endurance exercise session in the exercise intervention, and might not represent the baseline condition. Comparing the changes between the diet and exercise interventions might be confounded by a different response to acute exercise, although the conditions were similar pre‐ and post‐intervention. The results from the control group revealed no marked alterations in macrophage expression, suggesting that the response to acute exercise were similar pre‐ and post‐intervention.

Strengths in this study includes human material, a detailed analysis of commonly used markers for immune cells in adipose tissue. We also analyzed complete sets of markers, as opposed to single markers, and critically evaluated their expression across several immune cells and adipocytes (Figs. 2 and 3). Other strengths in this study includes the comparison of three methods in gene expression analysis (microarray, RNA sequencing and RT‐PCR). Moreover, both interventions were strictly supervised with similar alterations in energy balance.

## Conclusion

In energy restricted overweight/obese subjects (body weight reduced by 5% during a 12 weeks intervention) there was enhanced lipolysis as monitored by mRNA sequencing in adipose tissue and by plasma concentration of FFA. Furthermore, we observed increased expression of M1‐like macrophage genes in adipose tissue. In contrast, adipose tissue macrophages and T‐cell‐related gene expression was reduced after exercise in overweight/obese subjects along with reduced expression of M2‐specific macrophage genes. Both energy restriction and enhanced physical exercise affect energy‐related pathways as well as inflammatory processes in different ways in overweight/obese subjects, especially with respect to the different types of macrophages involved.
